# Family Voices in Digital Patient Navigation for Cervical Cancer Care in Indonesia

**DOI:** 10.3390/healthcare14131809

**Published:** 2026-06-23

**Authors:** Hana Rizmadewi Agustina, Hartiah Haroen, Tuti Pahria, Gatot Nyarumenteng Adhipurnawan Winarno, Citra Windani Mambang Sari, Windy Natasya, Heni Nur Anina, Inggriane Puspita Dewi, Yovita Dwi Setiyowati, Diwa Agus Sudrajat, Sita Sharma, Chyntya Putri Alita, Finny Fauziah Hidayat

**Affiliations:** 1 Department of Fundamental Nursing, Faculty of Nursing, Universitas Padjadjaran, Bandung 45363, West Java, Indonesia; 2 Department of Community Health Nursing, Faculty of Nursing, Universitas Padjadjaran, Bandung 45363, West Java, Indonesia; hartiah@unpad.ac.id (H.H.); citra.windani@unpad.ac.id (C.W.M.S.); 3 Department of Medical-Surgical Nursing, Faculty of Nursing, Universitas Padjadjaran, Bandung 45363, West Java, Indonesia; tuti.pahria@unpad.ac.id; 4 Dr. Hasan Sadikin General Hospital, Bandung 40161, West Java, Indonesia; gatot.nyarumenteng@unpad.ac.id (G.N.A.W.); natasyawindy@gmail.com (W.N.); heninuranina@gmail.com (H.N.A.); 5 Faculty of Medicine, Universitas Padjadjaran, Bandung 45363, West Java, Indonesia; inggriane23001@mail.unpad.ac.id; 6 Nursing Department, Faculty of Health Sciences, Universitas Aisyiyah, Bandung 40264, West Java, Indonesia; 7 Faculty of Nursing, Universitas Padjadjaran, Bandung 45363, West Java, Indonesia; yovitads@gmail.com (Y.D.S.); diwa.sudrajat@gmail.com (D.A.S.); chyntya23001@mail.unpad.ac.id (C.P.A.); finny21001@mail.unpad.ac.id (F.F.H.); 8 Nursing Department, STIK Sint Carolus, Jakarta 10440, DKI Jakarta, Indonesia; 9 Nursing Department, STIKep PPNI, Bandung 40173, West Java, Indonesia; 10 School of Nursing and Midwifery, University of Southern Queensland, Brisbane 4350, Australia; sita.sharma@unisq.edu.au

**Keywords:** cervical cancer, digital health, family, patient navigation, patient-centered care, health belief model, Indonesia

## Abstract

**Background**: Cervical cancer remains a significant health issue in Indonesia, where structural barriers, fragmented information, and sociocultural norms continue to hinder timely diagnosis and treatment. Families play a central role throughout the illness journey, yet their perspectives are often overlooked in the development of digital patient navigation systems. This study explored family experiences, caregiving challenges, and expectations for a family-centered digital navigation model, DIVA.ID, by integrating Digital Health frameworks and Family Systems Theory. **Methods**: A qualitative descriptive approach was employed through semi-structured, in-depth interviews with 18 purposively selected family caregivers of women with cervical cancer at a major referral hospital in West Java. Participants were selected because they were directly involved in daily care, treatment decisions, logistical support, or emotional assistance. Interviews were conducted between August and October 2025 and continued until thematic saturation was reached, as indicated by repetition of categories and the absence of new major codes in the final interviews. Data were analyzed using inductive–deductive content analysis guided by Elo and Kyngäs, with five researchers conducting independent coding, iterative code comparison, consensus meetings, and theoretical mapping. **Results**: Four main themes emerged: (1) family involvement in decision-making, including collective discussion, shifting authority roles, and patient autonomy; (2) caregiver burden, involving physical exhaustion, psychological distress, social restriction, stigma, financial pressure, and employment disruption; (3) psycho-spiritual coping mechanisms, including emotional sharing, prayer, crying, patience, and surrender to God; and (4) digital healthcare needs, covering BPJS guidance, treatment information, scheduling, communication pathways, shelter support, and mental–spiritual support. Mapping these themes to Digital Health frameworks and Family Systems Theory clarified how DIVA.ID could translate family experiences into practical navigation functions. **Conclusions**: This study provides empirical foundations for a culturally sensitive, family-centered digital navigation model in Indonesia. Rather than demonstrating effectiveness, the findings identify design requirements for DIVA.ID that should be tested in subsequent feasibility, usability, and intervention studies.

## 1. Introduction

Cervical cancer remains a significant global health challenge. According to the Global Cancer Observatory (GLOBOCAN), there were 662,301 new cases and 348,874 deaths worldwide in 2022, making it the fourth-most common cancer among women globally [1]. These updated figures show that cervical cancer remains a major public health issue despite the availability of HPV vaccination and screening technologies.

In Indonesia, the burden is even greater. In 2022, Indonesia reported 36,964 new cervical cancer cases and 20,708 deaths, with an incidence rate of 23.3 per 100,000 women, nearly twice the global average [2]. Screening uptake remains insufficient, and many women present at advanced stages because of restricted access, economic difficulties, limited diagnostic infrastructure, and cultural barriers surrounding discussions of reproductive health [3,4]. These conditions highlight the urgency of interventions that are accessible, affordable, and sensitive to the sociocultural realities of Indonesian women and their families.

The family context is particularly important in Indonesia. Family caregivers provide emotional, practical, financial, and decisional support for patients with cancer, and they often shape health-seeking behavior, treatment adherence, psychosocial adjustment, and long-term care planning [5]. In advanced care planning in Indonesia, families may influence patient autonomy and care trajectories, making them indispensable partners rather than peripheral supporters in cancer care [6]. However, this central family role is not yet adequately reflected in most digital patient navigation approaches.

Patient navigation (PN), originally developed by Harold Freeman, aims to remove barriers to timely cancer diagnosis and treatment for underserved populations [7]. The PN field has expanded globally, with evidence showing benefits across screening, diagnosis, treatment, survivorship, and palliative care [8]. The World Health Organization has similarly emphasized navigation as a strategy for improving care coordination, reducing delays, and supporting patients through complex cancer pathways [9]. Navigation is especially relevant for populations experiencing structural, cultural, and financial barriers, but its implementation must be adapted to local systems and navigator roles [10,11].

Most existing navigation models, however, conceptualize the primary user as an individual patient. This orientation is insufficient in collectivist settings such as Indonesia, where decisions about diagnosis, treatment, referrals, financing, and caregiving are commonly negotiated within the family. Family Systems Theory (FST) provides a useful lens for understanding this context because it views the family as an interconnected emotional unit in which illness in one member affects the whole system [12]. Evidence on FST has highlighted the relevance of differentiation, emotional interdependence, relational roles, and intergenerational patterns in explaining how families respond to chronic illness and stress [13,14,15].

The rapid growth of digital health technologies creates an opportunity to combine navigation with family-centered care. Digital health applications in oncology can enhance access, continuity, communication, and coordination when they are designed around user needs and health system constraints [16,17]. In Indonesia, where geographical distance, uneven specialist distribution, administrative complexity, and fragmented information persist, a digital platform may help families anticipate the care pathway, obtain reliable information, and communicate more effectively with services.

Despite this potential, the literature has not sufficiently synthesized digital patient navigation with family-centered care in the Indonesian cervical cancer context. The specific gap addressed in this study is therefore not simply the lack of another digital tool, but the lack of empirical evidence on how families themselves understand navigation problems and what functions they expect from a digital system. This distinction is central to DIVA.ID: the model is intended to treat family members as co-users and co-navigators rather than as informal bystanders.

Conceptually, DIVA.ID is expected to differ from generic digital navigation systems by incorporating four family-centered components: shared decision-support information for patients and relatives; practical navigation for BPJS procedures, scheduling, referrals, and treatment preparation; communication pathways linking families with clinical information and support resources; and culturally acceptable psycho-spiritual support, including motivation, emotional coping, and religiously sensitive content. These components are grounded in the view that navigation in Indonesia is simultaneously informational, relational, logistical, and spiritual.

Accordingly, this study aimed to explore family experiences, caregiving challenges, and expectations for a family-centered digital navigation system for women with cervical cancer in Indonesia. Specifically, the study sought to identify family roles in decision-making across the cervical cancer care pathway, describe caregiving burdens and psycho-spiritual coping mechanisms experienced by family members, examine family expectations for digital health support, including administrative, clinical, logistical, communication, and psychosocial needs, and translate these empirical findings into preliminary design requirements for the DIVA.ID digital navigation model.

## 2. Methods

### 2.1. Design

This study used a qualitative descriptive design based on semi-structured interviews with family caregivers of women with cervical cancer [18]. A qualitative descriptive approach was appropriate because the study sought to obtain a direct, practice-oriented account of family experiences, barriers, coping strategies, and expectations for digital navigation while remaining close to participants’ language and everyday realities.

### 2.2. Participants and Setting

The study was conducted at a major referral hospital in West Java that provides cervical cancer detection, diagnosis, treatment, and psychosocial support. The hospital was selected because families attending this setting frequently encounter complex treatment pathways, referral procedures, BPJS administrative requirements, travel demands, and prolonged treatment schedules, making it an information-rich site for exploring navigation needs.

Eighteen family members who were directly involved in the care of women with cervical cancer were purposively selected. Purposive sampling was used to capture caregivers who had first-hand experience in at least one of the following roles: accompanying patients during treatment, assisting with administrative procedures, participating in treatment-related decisions, providing emotional support, or coordinating household and financial responsibilities. Sampling also sought variation in relationship to the patient, gender, age, educational background, occupation, and duration of caregiving.

Eligible participants were family members aged 16 years or older who lived with or regularly supported the patient and voluntarily agreed to participate. For participants under 18 years of age, parental or guardian consent was obtained. Family members who were healthcare professionals or who had communication difficulties that prevented participation in an interview were excluded. All participants provided written informed consent before data collection.

Sample adequacy was assessed through thematic saturation and informational sufficiency. During data collection and preliminary analysis, the research team monitored whether new interviews produced new codes, new sub-themes, or substantially different interpretations relevant to the study objectives. By the fifteenth interview, the major categories had become stable; three additional interviews were conducted to confirm repetition across family roles and caregiving durations. Interviews were concluded after no new major categories emerged, and the existing themes were judged sufficiently rich to support theoretical mapping.

### 2.3. Data Collection

Data were collected between August and October 2025 through semi-structured, in-depth interviews with family members of women diagnosed with cervical cancer. Interviews were conducted face-to-face in private rooms at the hospital to ensure comfort and confidentiality. Each interview lasted approximately 45–60 min and was audio-recorded with participants’ permission.

The interview guide explored family members’ experiences in providing emotional and practical support, participation in treatment decisions, communication with healthcare providers, challenges during treatment, use of informal information sources, and expectations toward a digital navigation program such as DIVA.ID. The guide was used flexibly so that participants could elaborate on issues they considered important, including BPJS procedures, treatment scheduling, accommodation, transportation, stigma, and spiritual coping. All interviews were conducted in Bahasa Indonesia, transcribed verbatim, and checked against the audio recordings for accuracy.

### 2.4. Data Analysis

Data were analyzed using qualitative content analysis as described by Elo and Kyngäs [19]. The analysis involved three iterative phases. In the preparation phase, transcripts were read repeatedly to gain immersion in the data. In the organization phase, meaning units were identified, open codes were assigned, and related codes were grouped into sub-themes and themes. In the reporting phase, categories were synthesized into a coherent account of family experiences, needs, and implications for DIVA.ID.

The analysis used a hybrid inductive–deductive strategy. Inductively, codes were generated from participants’ narratives to preserve context-specific meanings. Deductively, emerging categories were compared with concepts from Digital Health frameworks, including navigation functions, usability, accessibility, communication, and informational continuity, and with Family Systems Theory constructs, including family roles, emotional interdependence, stress processes, and coping [12,16,17]. This approach allowed the analysis to remain grounded in data while clarifying how empirical findings could inform a theoretically coherent digital navigation model.

Five researchers independently coded selected transcripts using Taguette (Version 1.5.1), which supported systematic coding, retrieval, categorization, and theme development [20]. Coding reliability was strengthened through structured code comparison rather than by calculating a numerical inter-coder reliability coefficient, which is not always appropriate for interpretive qualitative content analysis. Each coder first developed provisional codes independently. The team then compared code definitions, discussed disagreements, refined the codebook, and reached consensus on final categories. Discrepancies were resolved through discussion focused on the meaning of the source text, fit with the developing codebook, and relevance to the study objectives.

**Figure 1:**
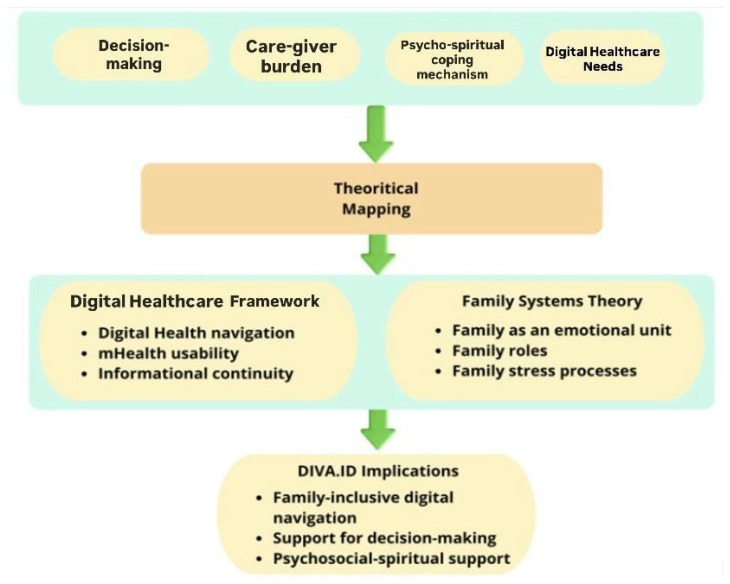
Mapping qualitative findings to theoretical frameworks and design implications for DIVA.ID. The figure summarizes how empirical themes were first derived from interviews, then interpreted through Digital Health frameworks and Family Systems Theory, and finally translated into candidate navigation functions. The mapping is intended to support model development and does not represent an evaluation of DIVA.ID effectiveness.

The conceptual mapping presented in Figure 1 was constructed in three steps. First, empirical themes were derived from coded data. Second, each theme was aligned with relevant theoretical domains from Digital Health frameworks and Family Systems Theory. Third, implications for DIVA.ID were specified by translating the themes into candidate system functions. The mapping is therefore interpretive and design-oriented; it does not imply tested causal relationships or proven system effectiveness.

### 2.5. Trustworthiness

Trustworthiness was established using Lincoln and Guba’s criteria of credibility, dependability, confirmability, and transferability [21]. Credibility was enhanced through member checking of key interpretations with participants when feasible, peer debriefing among the research team, and comparison of themes across different family relationships. Dependability was supported by documenting recruitment decisions, interview procedures, coding steps, codebook revisions, and consensus meetings in an audit trail. Confirmability was strengthened through reflexive journaling, in which researchers recorded assumptions, emerging interpretations, and potential sources of bias, particularly because several researchers were familiar with oncology and nursing contexts. Transferability was supported by providing detailed descriptions of the setting, participant characteristics, family roles, and caregiving context so that readers can judge the applicability of the findings to similar referral hospitals and collectivist care settings.

## 3. Results

### 3.1. Participants’ Characteristics

This study involved 18 family members of women diagnosed with cervical cancer who provided ongoing caregiving support during treatment. Participants included nine males and nine females, aged 16–54 years. Most were husbands (33%) or daughters (28%), with the remainder comprising sons, the patient’s mother, cousins, and a niece. Educational levels ranged from primary school to bachelor’s degree, with many participants having completed junior high school. Most were self-employed or unemployed, and caregiving duration ranged from one month to one year. Overall, the sample represented family members who were directly involved in emotional support, administrative navigation, transport, treatment accompaniment, and household adjustment (Table 1).

### 3.2. Findings and Analysis

#### 3.2.1. Findings

The qualitative findings reveal how families navigate decision-making, caregiving burden, coping, and digital support needs during cervical cancer care. Four themes and their refined sub-themes are summarized in Table 2. The refinement is especially important for the theme of digital healthcare needs, which was broad in the earlier version and is now differentiated into concrete informational, administrative, logistical, communication, and psycho-spiritual needs.

#### 3.2.2. Analysis

A hybrid content analysis approach was employed, integrating both deductive and inductive strategies. Deductively, an initial coding matrix was developed using predefined categories from Digital Health frameworks, including navigation functions, usability, accessibility, communication, and informational continuity, and from Family Systems Theory, including family roles, emotional interdependence, and stress processes [12,16,17,22]. These theory-derived categories provided a guiding structure for organizing the data. Inductively, the analysis allowed new categories to emerge directly from participants’ narratives, ensuring that contextual nuances, unmet digital needs, caregiving pressure, and psycho-spiritual coping were retained.

The qualitative findings show that families do not experience cervical cancer care as a purely clinical pathway. Instead, they experience it as a relational, financial, administrative, geographical, and spiritual process. Decision-making was often shared across family members, with authority shifting according to age, knowledge, proximity to the patient, and the absence of other decision-makers. Caregiver burden extended beyond physical fatigue to include emotional vigilance, social withdrawal, stigma, transport expenses, and work disruption. Coping was strongly psycho-spiritual, as participants used prayer, crying, patience, surrender, and emotional disclosure to preserve resilience.

Figure 1 illustrates how these themes were mapped to the two theoretical frameworks and then translated into design implications. The left side of the figure represents empirical themes derived from the interviews. The middle layer shows the theoretical domains used for interpretation: Digital Health domains explain information flow, access, usability, and continuity, whereas Family Systems Theory explains relational roles, emotional interdependence, shared decision-making, and stress regulation. The right side summarizes the resulting implications for DIVA.ID. The arrows therefore represent analytical translation from data to theory to design, not causal pathways tested by this qualitative study (Table 3).

## 4. Discussion

This study provides an empirically grounded account of how Indonesian families experience cervical cancer care and what they expect from a digital navigation system. The findings extend the discussion of patient navigation by showing that, in this context, navigation is not only an individual patient function but also a family system function [23]. Families were deeply involved in deciding, accompanying, financing, interpreting information, coping emotionally, and sustaining care. These findings support the development of DIVA.ID as a family-centered digital navigation model that is responsive to local barriers while remaining cautious about claims of effectiveness before intervention testing.

Family involvement in decision-making was central to participants’ accounts. Decisions were rarely described as isolated individual choices; rather, they were negotiated through family discussions, caregiver authority, patient preferences, and practical constraints. This pattern is consistent with evidence that families in Indonesia shape cancer care planning and patient autonomy [6]. From the perspective of Family Systems Theory, the illness of one family member reorganizes the whole family system, redistributing roles, emotional responsibilities, and decision-making authority [12,13,14,15]. In the DIVA.ID model, this finding implies that navigation information should be understandable not only to patients but also to spouses, adult children, siblings, and other relatives who participate in care decisions [23,24].

Caregiver burden was multifaceted and extended beyond the time spent accompanying patients. Participants described physical fatigue from repeated treatment visits, psychological distress from constant vigilance, social restriction due to hospital-based caregiving, stigma from neighbors, transport costs, daily living expenses, and loss of income or employment. These findings align with Indonesian evidence showing a substantial burden among caregivers of women with cancer [5]. The practical implication is that DIVA.ID should not be limited to biomedical information [23,25]. It should include tools that reduce administrative uncertainty, anticipate treatment schedules, clarify BPJS procedures, and help families prepare for transport, accommodation, and daily care needs.

Psycho-spiritual coping emerged as a prominent mechanism through which families managed distress. Participants described crying, prayer, patience, surrender to God, and sharing stories with trusted people [26,27]. These strategies should not be treated as peripheral cultural details. They indicate the form of support that may be acceptable, meaningful, and sustainable for families. For DIVA.ID, this suggests the need for optional psycho-spiritual and motivational resources that complement clinical navigation. Such features should be carefully designed to avoid replacing professional mental health care, but they may help normalize emotional distress, encourage caregiver self-care, and connect families to appropriate support when needed [23,28].

The digital healthcare needs theme provides the clearest bridge between empirical data and system design. Families requested practical, immediate, and locally relevant information: BPJS guidance, registration and scheduling support, treatment and medication information, home-care advice, dietary guidance, hospital contact pathways, shelter locations, and psychological or spiritual encouragement. These expectations are consistent with the broader promise of digital health in improving access, coordination, communication, and continuity of oncology care [16,17]. Evidence from previous cervical cancer screening and digital health interventions that informed the conceptual development of DIVA.ID is summarized in the Supplementary Materials [29,30,31,32]. However, the findings also show that digital navigation should be simple, affordable, and usable across different educational and digital literacy levels [33].

The recommendations for DIVA.ID therefore arise directly from the data. First, DIVA.ID should include shared decision-support content that explains the cervical cancer care pathway in language accessible to families [24]. This recommendation is grounded in the theme of family involvement in decision-making. Second, it should provide practical navigation modules for BPJS, registration, scheduling, treatment preparation, and accommodation. This recommendation reflects caregivers’ reported financial and logistical burden. Third, it should include verified communication pathways and clinical update mechanisms to reduce reliance on fragmented informal information [34,35]. Fourth, it should offer optional psycho-spiritual and motivational support to reflect families’ culturally grounded coping practices. These functions should be developed through participatory co-design with patients, caregivers, nurses, physicians, navigators, and hospital administrators.

The study also contributes conceptually by integrating Digital Health frameworks with Family Systems Theory. Digital Health frameworks explain how a platform can improve information continuity, usability, coordination, and access [22]. Family Systems Theory explains why the platform must be family-inclusive in contexts where relatives influence care trajectories. Together, these frameworks suggest that digital navigation should be designed not merely as a technical information channel but as a relational infrastructure that supports families as co-navigators across the cancer care continuum. This is the main conceptual distinction between DIVA.ID and individual-oriented digital navigation tools.

At the same time, the findings should be interpreted within the scope of a qualitative descriptive study. The study identifies needs, expectations, and design requirements; it does not test whether DIVA.ID improves care coordination, treatment adherence, psychological outcomes, or continuity of care. Claims about effectiveness should therefore be reserved for future feasibility, usability, pilot, and controlled intervention studies. The present findings provide the empirical and theoretical basis for such future work.

### Strengths and Limitations

This study’s strength lies in its combination of rich qualitative data with Digital Health frameworks and Family Systems Theory, which enabled the analysis to connect lived family experiences with concrete design implications. The study also included different caregiver roles, including husbands, daughters, sons, cousins, a niece, and the patient’s mother, allowing the analysis to capture variation in authority, proximity, and responsibility within family systems.

Several limitations should be acknowledged. First, the study was conducted in a single referral hospital in West Java, and the findings may not fully represent families in primary care, rural facilities, private hospitals, or other Indonesian regions with different health system pathways. Second, purposive sampling enabled the selection of information-rich participants but may have introduced selection bias, because families willing to be interviewed may differ from those experiencing greater distress, conflict, or digital exclusion. Third, social desirability bias may have influenced participants to emphasize socially acceptable forms of family support, patience, or religious coping. Fourth, researcher influence is possible because interviewers and analysts were connected to health and nursing contexts, although reflexive journaling and team discussions were used to reduce this risk. Fifth, the study included family perspectives but did not directly examine patients’, clinicians’, hospital administrators’, or technology developers’ views. Finally, participants’ digital literacy varied, and this may shape the feasibility and usability of any digital navigation model [36]. Future research should therefore include multi-center samples, participatory co-design, usability testing, and intervention evaluation.

## 5. Conclusions

This study suggests that Indonesian families play a crucial role in cervical cancer care by shaping decision-making, sustaining caregiving work, managing logistical and financial challenges, and using psycho-spiritual strategies to cope with uncertainty and distress. Persistent barriers, including limited screening access, fragmented information, administrative complexity, logistical strain, and stigma, make family-centered navigation especially relevant in the Indonesian context.

By aligning qualitative findings with Digital Health frameworks and Family Systems Theory, the study identifies design requirements for DIVA.ID as a culturally sensitive, family-inclusive digital navigation model. The findings support the inclusion of shared decision-support information, BPJS and scheduling guidance, treatment and home-care education, communication pathways, accommodation support, and psycho-spiritual resources. These conclusions should be understood as design-oriented inferences from qualitative data rather than evidence of DIVA.ID effectiveness. Future studies should evaluate the acceptability, usability, feasibility, and clinical or psychosocial impact of DIVA.ID before claims of improved outcomes are made.

## Figures and Tables

**Table 1:** Characteristics of Participants (n = 18).

Codes	Age (Years)	Gender	Last Education	Occupation	Family Relation	Duration of Care
K1	40	Male	Junior High School	Self-employed	Husband	2 months
K2	16	Female	Senior High School	Unemployed	Daughter	2 months
K3	35	Female	Primary School	Private employee	Daughter	5 months
K4	32	Female	Junior High School	Housewife	Mother	1 month
K5	48	Male	Senior High School	Self-employed	Husband	1 year
K6	34	Male	Vocational High School	Unemployed	Husband	1 year
K7	31	Female	Bachelor’s degree	Teacher	Daughter	1 year
K8	54	Female	Senior High School	Unemployed	Daughter	5 months
K9	53	Male	Junior High School	Self-employed	Husband	1 year
K10	18	Male	Senior High School	Freelancer	Son	2 months
K11	48	Male	Primary School	Self-employed	Husband	2 months
K12	27	Male	Vocational High School	Self-employed	Son	2 months
K13	51	Male	Junior High School	Self-employed	Husband	1 year
K14	40	Female	Junior High School	Event organize	Cousin	6 months
K15	38	Female	Junior High School	Housewife	Cousin	2 months
K16	29	Female	Bachelor’s degree	Unemployed	Niece	1 year
K17	24	Male	Vocational High School	Employee	Son	1 year
K18	25	Female	Senior High School	Self-employed	Daughter	5 months

Note. The table was reformatted for readability; participant codes and demographic information are retained.

**Table 2:** Themes, refined sub-themes, and illustrative quotes from family members’ perspectives.

Themes	Sub-Themes	Quotes
Family Involvement in Decision-Making	Family group conference	“I need to discuss it first with my parents and family as well; the decision has to be medical.” (K1) “This problem should be solved through family discussion.” (K9) “...a family discussion... If you want to get better, there must be an answer” (K13)
	Authority of the patient’s family member	“When it comes to making decisions, it’s up to me… my father doesn’t understand, so I’m the one who makes all the decisions for my mother.” (K3) “Father has passed away... My older brother and I were the ones who made the decision.” (K10)
	Patient autonomy within family consultation	“Mama is the one who decides everything… sometimes she discusses it with the shelter.” (K2)
Caregiver Burden	Physical exhaustion	“Stress, tiredness... the exhausting journey of daily radiotherapy for 25 days.” (K3) “For me personally, it’s the physical issues. It’s obvious since we are taking care of sick people, it’s exhausting to manage because we worry about their condition.” (K6)
	Psychological distress and constant vigilance	“I feel like my world is falling apart, like being bombed!!... But we are in front of the family, so we have to stay strong, right?” (K7) “Yes, it’s stressful, ma’am. I want to... take turns caring for them. If I go get something to eat... there’s no one to accompany the patient. If a doctor comes... there’s no one to accompany them.” (K14)
	Social restriction	“I have to stay here. My child was entrusted to my sister-in-law...” (K3)
	Cancer-related stigma	“My neighbors often say, ‘If you get cancer, you won’t recover; many have died from it.” (K7)
	Transportation, living costs, and patient needs	“The only problem is transportation. BPJS doesn’t cover the travel expenses, so we have to pay again. The patient’s treatment is covered, but the family members who accompany still have additional and unexpected expenses.” (K9) “Yes, it’s the transportation cost. At home, we also have other expenses, like buying diapers. Yes, financially, that’s the issue, sir.” (K11)
	Loss of work and assets	“I used to work, but I resigned so I could take care of my mother.” (K2) “The financial issue is also difficult. We had to rent vehicles, hire cars for transport.” (K12)
Psycho-spiritual coping mechanism	Sharing a story with a friend or spouse	“Sometimes, we just need someone to talk to, so I just tell my story. It’s better to talk about it than to keep everything inside.” (K17)
	Crying, praying, and surrendering to God	“Just pray… sometimes someone reminds me to pray, even at night, around two in the morning.” “If I keep thinking about it, I’ll just get sick, so I’ll let it go. Earlier, I cried…” (K4) “Sometimes I feel up and down, almost hopeless, but if we want recovery, we must stay patient. So, yes… just keep praying.” (K13)
Digital healthcare needs	Perceived usefulness, preparedness, affordability, and quality	“It’s very helpful, especially for those coming from outside Bandung. Really helpful. Yes, it’s needed.” (K8) “I agree, ma’am… it’s important to know things beforehand so we can be prepared.” (K14) “Yes, it’s necessary, if possible. It should be free, but with good quality.” (K15) “Yes, I think it’s needed, because for me, I look everything up on the internet.” (K16)
	Information about accommodation and shelters near the hospital	“There are many kinds of shelter homes, some are good, some are not. I also looked for a rented place that would be comfortable for my mother.” (K8) “A location point for those living in shelters would be useful.” (K12)
	BPJS, administration, registration, and scheduling guidance	“For example, information related to BPJS (Government Health Insurance) could be included.” (K5) “Like, for example, registration, scheduling, things like that.” (K7)
	Treatment pathway, medication, disease progression, diet, and home-care information	“Medication, restrictions, and information would be easier to access.” (K3) “From early management to the end, until home care is possible, including diet and herbal treatments.” (K6)
	Communication channels and clinical updates	“WhatsApp contacts, patient conditions, and treatment updates.” (K1)
	Psychological, motivational, and spiritual support	“What we hope for the most is motivation and encouragement.” (K11) “There should be a feature or section to provide mental or religious support.” (K12)

Note. The digital healthcare needs theme was expanded into more specific sub-themes to clarify how families expect DIVA.ID to function in practice.

**Table 3:** Operational translation of empirical themes into preliminary DIVA.ID design features.

Empirical Theme	Design Requirement	Candidate DIVA.ID Functionality	Rationale Grounded In Findings
Family involvement in decision-making	Support collective and role-shifting decision processes	Family-accessible decision-support pages; treatment pathway explanations; question prompts for family discussion	Families described decisions as negotiated through group discussion, caregiver authority, and patient autonomy.
Caregiver burden	Reduce logistical, administrative, and informational strain	BPJS step-by-step guide; appointment and treatment schedule tracker; checklist for radiotherapy/chemotherapy preparation; transport and cost-planning information	Caregivers reported exhaustion, fragmented information, transport costs, administrative difficulty, and competing household responsibilities.
Psycho-spiritual coping mechanisms	Provide culturally acceptable emotional and spiritual support	Motivational messages; coping resources; links to psychological support; optional religious/spiritual content; caregiver self-care prompts	Participants relied on prayer, patience, surrender, crying, and emotional sharing to cope with uncertainty and distress.
Digital healthcare needs	Convert broad digital expectations into usable navigation modules	Shelter/accommodation information and map; verified hospital contacts; treatment, medication, diet, and home-care information; communication pathway for updates	Families requested practical information on BPJS, registration, scheduling, shelter location, treatment restrictions, home care, and mental–spiritual support.

Note. Table 3 presents design requirements generated from this qualitative study. These features require further co-design, usability testing, feasibility assessment, and effectiveness evaluation before implementation.

## Data Availability

The data supporting the findings of this study are not publicly available due to ethical considerations and participant privacy protection.

## Supplementary Materials

The following supporting information can be downloaded at: https://www.mdpi.com/article/10.3390/healthcare14131809/s1.
