# Supplementary material for: Family Voices in Digital Patient Navigation for Cervical Cancer Care in Indonesia
Source: Healthcare (Basel). 2026 Jun 23;14(13):1809. doi: 10.3390/healthcare14131809 (PMC13362353; doi:10.3390/healthcare14131809)
Supplement: Supplementary file 1 [file healthcare-14-01809-s001.zip › healthcare-4320951-supplementary.pdf]

No	Author(s)	Year of Publication	Country	Objectives	Sample	Method	Intervention	Key Findings
1	Mboineki et al.	2024	Tanzania	Assess effect of patient navigation on cervical cancer screening.	202 community women randomized (101 intervention, 101 control).	Randomized Controlled Trial	Patient navigation (education, scheduling, counseling).	Screening uptake increased significantly in intervention group, participants showed higher knowledge scores, reduced perceived barriers, and improved health beliefs toward cervical cancer screening.
2	Mboineki et al.	2022	Tanzania	Evaluate peer-led navigation for screening uptake.	44 women (intervention vs control groups).	Randomized Controlled Trial	Peer-led navigation via community health workers.	Screening rate reached 72.7% in intervention vs very low in control, peer support improved trust, motivation, and accessibility to services.
3	Lee et al.	2024	Malawi	Evaluate mHealth storytelling intervention.	179 women with HIV (60, 59, 60 per group).	Pilot RCT	Mobile video-based narrative intervention.	Intervention groups showed significantly higher screening uptake, increased awareness, improved risk perception, and stronger intention to undergo screening.
4	Okunade et al.	2021	Nigeria	Assess effect of mHealth SMS on Pap smear uptake.	Women in 2 tertiary hospitals (RCT sample).	Randomized Controlled Trial	SMS reminders for screening.	Significant increase in Pap smear attendance, reminders reduced forgetfulness, improved appointment adherence, and encouraged proactive health behavior.

5	Arrossi et al.	2019	Argentina	Improve follow-up of HPV-positive women.	Women with HPV self-collection in community settings.	Cluster RCT	SMS + phone-based mHealth follow-up.	Follow-up adherence significantly improved; the intervention reduced loss-to-follow-up and enhanced continuity of care after initial screening.
6	Zhang et al.	2024	China	Evaluate the online cervical cancer screening system.	Women participating in a national online screening trial (community-based sample).	Experimental study	Online platform + self-sampling HPV test.	The online system improved access to screening, increased participation, especially in remote areas, and reduced logistical barriers such as distance and hospital visits.
7	Straw, C., Sanchez-Antelo, V., Kohler, R. et al.	2023	Argentina	Evaluate the scaling of the mHealth intervention.	HPV-positive women in the public health system.	Implementation study	SMS reminders + CHW follow-up.	Improved adherence to triage after a positive HPV test and reduced loss to follow-up in screening programs.
8	Ssedyabane et al.	2024	Uganda	Develop an mHealth intervention to reduce loss to follow-up.	Patients are undergoing treatment for cervical lesions in a referral hospital.	Qualitative + intervention development	Customized mHealth system.	Identified barriers (transport and communication gaps) and implemented a tailored digital system, improving continuity of care and patient tracking.
9	Ahadi et al	2025	Iran	Compare mHealth vs face-to-face education.	Women in Saveh (parallel RCT groups).	Randomized Controlled Trial	Mobile app education vs F2F training.	mHealth significantly improved knowledge, attitude, self-efficacy, and intention to perform a Pap smear compared to traditional education.
10	Razzak et	2023	Bangladesh	Evaluate the usability of	10 mobile apps	Mixed-method	mHealth app	Found major usability issues

	al.			mHealth apps for cervical cancer.	evaluated by 4 usability experts.	(heuristic + user evaluation)	evaluation.	(navigation, clarity, feedback) that reduce user engagement, and poor design can limit the effectiveness of digital interventions.
11	Wang J et al.	2022	Europe (multi-country)	To evaluate the feasibility of risk-stratified cervical cancer screening.	Women aged 25–65 participating in pilot cervical screening programs in multiple European countries (exact n not specified).	Pilot study	Risk-stratified screening.	Risk-based screening improved the identification of high-risk individuals and optimised screening efficiency compared to conventional uniform approaches.
12	Fujita, M., Nagashima, K., Shimazu, M. et al.	2022	Japan	To assess the effectiveness of HPV self-sampling among non-responders.	1,206 women aged 30–59 years who did not attend routine cervical screening in Japan.	Randomized Controlled Trial	HPV self-sampling.	Self-sampling significantly increased screening uptake among non-attenders by reducing embarrassment, time constraints, and access barriers.
13	Sultanov, M., Zeeuw, J.D., Koot, J. et al.	2022	Multi-country (India, Bangladesh, Uganda)	To evaluate the feasibility of the WHO cervical cancer screening protocol.	Women aged 30–49 years from community-based screening programs across LMIC settings (n ≈ 10,000+).	Implementation study	HPV screen-and-treat.	The WHO protocol was feasible and scalable, improving early detection and enabling timely treatment in low-resource settings.
14	Teigné et al.	2022	United Kingdom	To assess the impact of invitation strategies on	2,635 women aged 25–64 years are	Randomized trial	Invitation letters.	Personalized and targeted invitation letters increased

				screening uptake.	eligible for cervical screening in the UK.			screening participation. highlighting the importance of communication strategies.
15	Chan et al.	2023	Malaysia	To examine factors influencing the prevalence of cervical cancer screening among women in Malaysia.	5,650 women aged 18–65 years from a nationwide population-based survey in Malaysia.	Cross-sectional study		Screening uptake was low and influenced by education, knowledge, and access to healthcare. Women with greater awareness were more likely to be screened, and social and family support also contributed to participation.
